# Assessment of Hypercoagulability in Splanchnic Vein Thrombosis by Measurement of the Hemostasis Enzymes Thrombin and Activated Protein C

**DOI:** 10.3390/ijms26010292

**Published:** 2024-12-31

**Authors:** Sara Reda, Johannes Chang, Johanna Busse, Nadine Schwarz, Hannah L. McRae, Jens Müller, Christian P. Strassburg, Johannes Oldenburg, Bernd Pötzsch, Christian Jansen, Heiko Rühl

**Affiliations:** 1 Institute of Experimental Hematology and Transfusion Medicine, University Hospital Bonn, 53127 Bonn, Germany; sara.reda1@uk-koeln.de (S.R.); bu.johanna@web.de (J.B.); nadine.schwarz@ukbonn.de (N.S.); johannes.oldenburg@ukbonn.de (J.O.);; 2 Department of Transfusion Medicine, University Hospital Cologne, 50937 Cologne, Germany; 3 Department of Internal Medicine I, University Hospital Bonn, 53127 Bonn, Germanychristian.strassburg@ukbonn.de (C.P.S.); christian.jansen@ukbonn.de (C.J.)

**Keywords:** activated protein C, hypercoagulability, myeloproliferative neoplasms, splanchnic vein thrombosis, thrombin

## Abstract

Splanchnic vein thrombosis (SVT), which is particularly prevalent in myeloproliferative neoplasms (MPNs), has a multifactorial pathomechanism involving the anticoagulant protein C (PC) pathway. To better characterize the hypercoagulable state in SVT we assessed its key enzymes thrombin and activated PC (APC). The study population included 73 patients with SVT, thereof 36 MPN+, confirmed by bone marrow biopsy, 37 MPN−, and 30 healthy controls. Direct measurement of the active enzyme forms of thrombin and APC in the circulation was achieved by using oligonucleotide-based enzyme capture assays (OECA). Additionally, activation markers of coagulation and fibrinolysis were measured. Plasma levels of free thrombin and APC were higher in the MPN+ than in the MPN− cohort, with 0.49 vs. <0.46 pmol/L (*p* = 0.0057), respectively, 1.23 vs. 0.58 pmol/L (*p* = 0.0122), and in healthy controls (vs. <0.46 pmol/L, *p* = 0.0012; vs. 0.54 pmol/L, *p* = 0.0035). The indirect activation markers prothrombin fragment 1+2, thrombin-antithrombin complex, and D-dimer did not differ between groups. Receiver operating characteristic analysis suggested that SVT patients with MPN can be better distinguished by APC than by conventional indirect thrombin markers. A potential application of these biomarkers to guide anticoagulant therapy and to investigate the role of the PC pathway in MPN-associated hypercoagulability should be further studied.

## 1. Introduction

Splanchnic vein thrombosis (SVT) comprises thrombosis of the portal venous system, including portal vein thrombosis (PVT), mesenteric vein thrombosis (MVT), and splenic vein thrombosis, as well as thrombosis of the hepatic venous system, referred to as Budd–Chiari syndrome [1,2]. Compared with the typical manifestation of venous thrombosis, i.e., deep vein thrombosis (DVT) of the lower extremity with annual incidence rates of 1–2 per 1000 person-years [3], it is a rare disease. For PVT, the most common site of SVT, incidence rates of 2–4 per 100,000 person-years were reported, whereas the annual incidence of Budd–Chiari syndrome, the least common manifestation, was estimated to be 1–2 per million [4,5,6].

The etiology of SVT is heterogeneous. One major risk factor is the presence of myeloproliferative neoplasms (MPN). MPN are prevalent in approximately 10%, and the somatic Janus kinase 2 (JAK2) V617F mutation, which is strongly associated with MPN, in about one-third of patients with SVT [7,8]. Other common risk factors include non-hematologic malignancies and liver cirrhosis, with each of them being present in approximately a quarter of patients with SVT in unselected cohorts [7,9,10]. Less common risk factors include abdominal inflammation or surgery, hormonal influences, and isolated classical hereditary thrombophilia [9,10].

The pathomechanisms of hypercoagulability in patients with major risk factors of SVT, i.e., MPN, solid malignancies, and liver cirrhosis, vary, but the endothelial protein C (PC) system is involved in all three risk groups. In this anticoagulant pathway, thrombin formation induces formation of activated PC (APC) from PC on endothelial cells through an activation complex between thrombin, thrombomodulin, and the endothelial PC receptor (EPCR) [11,12]. After release into the circulation, APC downregulates thrombin formation through proteolytic inactivation of the activated factors V and VIII, enhanced by complex formation with its cofactor protein S (PS) [13,14]. Patients with MPN develop an APC resistance phenotype, and the expression of JAK2 V617F in endothelial cells is associated with further prothrombotic alterations [15,16]. In solid cancer, several mechanisms, including increased tissue factor expression, promote thrombin formation, while activation or damage of the endothelium may also contribute to hypercoagulability [17,18]. In liver cirrhosis, the PC system is impaired by an increased ratio of factor VIII versus PC and PS, resulting in increased thrombin generation [19,20].

Anticoagulation is the cornerstone of therapy in SVT, which has been traditionally conducted using heparin and vitamin K antagonists (VKA) [1,21]. Direct oral anticoagulants (DOACs) are increasingly used, but they are still off-label for patients with SVT and contraindicated in more severe liver cirrhosis [21,22]. After acute treatment of SVT, anticoagulation is continued in the presence of persistent risk factors and can also be considered in cases of unprovoked SVT [23,24]. Especially in patients with liver disease, which may also be caused or aggravated by SVT, anticoagulation is difficult due to an increased risk of bleeding and thrombosis [25,26]. Anticoagulant drugs also interfere with the PC system, as they eventually downregulate thrombin formation via different mechanisms of action, and, in the case of VKA, additionally reduce the plasma levels of PC and PS.

In view of the involvement of the PC system in the pathomechanism of SVT, the aim of this study was to better characterize the coagulopathy in patients with SVT by measuring the key enzymes of coagulation and the anticoagulant PC pathway, thrombin, and APC. We hypothesized that patients with different etiologies of SVT, i.e., with or without MPN, could be distinguished using these biomarkers. Furthermore, we aimed to assess the impact of anticoagulant therapy on the PC system in patients with SVT.

## 2. Results

### 2.1. Study Population

**Figure 1:**
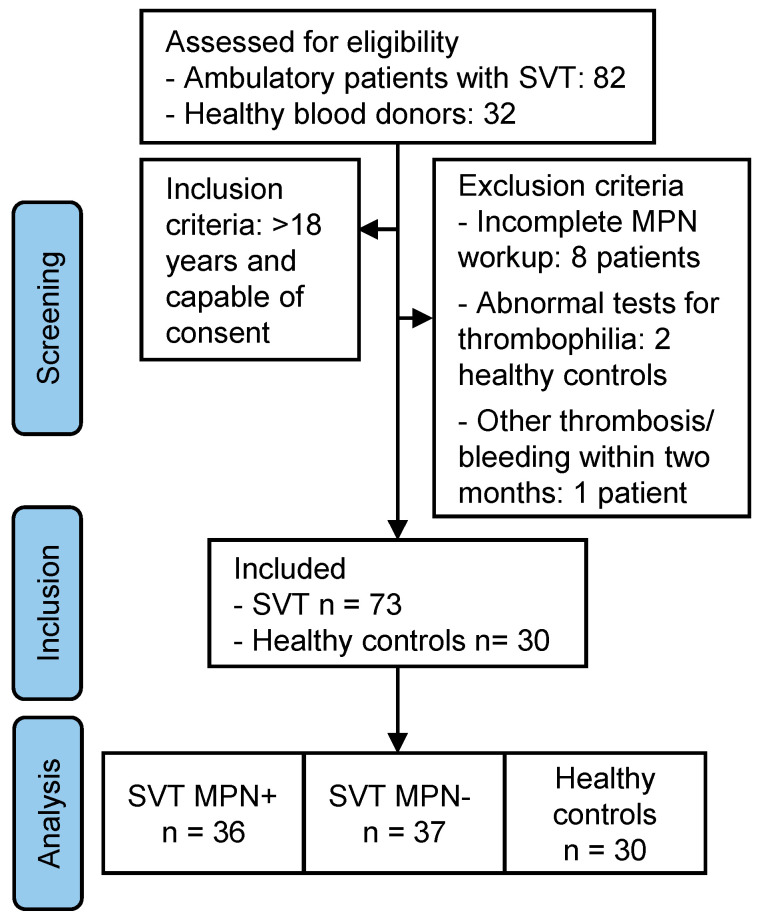
Study flow chart. Workup of myeloproliferative neoplasms (MPN) included complete blood count, peripheral blood smear review, molecular genetic analysis of MPN-associated clonal mutations, and, in the case of abnormalities, bone marrow biopsy. Thrombophilia screening included testing for deficiencies of antithrombin, protein C, protein S, factor V (FV) Leiden and prothrombin 20210G>A mutations, and antiphospholipid antibodies. Abnormalities in thrombophilia screening were an exclusion criterion only for healthy controls, of whom two were excluded due to FV Leiden mutation. Other thrombosis was defined as any arterial or venous thrombosis other than splanchnic vein thrombosis (SVT). Bleeding was defined as any bleeding event requiring medical treatment, and one patient was excluded due to recent lower gastrointestinal bleeding requiring blood transfusion.

The study population included 73 patients with SVT, thereof 36 in the SVT MPN+ cohort and 37 in the SVT MPN− cohort, and 30 healthy controls. A study flow chart is shown in Figure 1, along with a description of the selection criteria. Among the patients with MPN were 14 cases of polycythemia vera (PV), 9 cases of essential thrombocythemia (ET), 7 patients with primary myelofibrosis (PMF), 2 patients with post-PV myelofibrosis, and 4 patients with unclassifiable MPN. In only one patient in the SVT MPN+ cohort, no MPN-associated somatic mutation was found, and the diagnosis of ET was confirmed by immunohistochemistry of bone marrow biopsy in a reference laboratory. In one patient in the SVT MPN− group, repeated testing for JAK2 V617F in peripheral blood samples did not clearly yield positive or negative results. Eventually, a very low mutation burden was confirmed in the bone marrow but, overall, the criteria for MPN were not met.

Characteristics of the study population are shown in Table 1. Both patient cohorts did not differ in age, with a mean age (range) of 56 (29–80) years in the SVT MPN+ group, and 56 (32–72) years in the SVT MPN− group. However, owing to the upper age limit for blood donation, the healthy controls were younger, with 48 (29–67) years (*p* = 0.0082 compared with SVT MPN+, *p* = 0.0064 compared with SVT MPN−). The rate of male/female patients and the body mass index did not differ between the three cohorts. No statistically significant differences were observed regarding the localization of SVT, time between SVT diagnosis and blood sampling, prevalences of additional thromboses at other sites, particularly prior VTE, coronary artery disease, malignancy other than MPN, thrombophilia risk factors, arterial hypertension, diabetes mellitus, autoimmune disorders other than inflammatory bowel disease, recent abdominal surgery, and the rates of patients receiving anticoagulant or antiplatelet medication. Liver disease, inflammatory bowel disease, and other infection/inflammation, which are frequent comorbidities and provoking factors of SVT, were more prevalent in the SVT MPN− cohort than in the SVT MPN+ cohort, with 32% versus 3% (*p* = 9.3 × 10^−4^), 16% versus 0% (*p* = 0.0251), and 32% versus 11% (*p* = 0.0276), respectively. A total of 67% of the patients in the SVT MPN+ group and none in the SVT MPN− group were on cytoreductive therapy (*p* = 1.1 × 10^−10^).

The platelet and white blood cell (WBC) counts were higher in the SVT MPN+ cohort than in the SVT MPN− cohort, with median values of 308 vs. 201 × 10^9^/L (*p* = 1.8·10^−4^) and 7.9 vs. 5.8 × 10^9^/L (*p* = 0.0042), respectively. Compared with healthy controls (median 255 × 10^9^/L), the platelet counts in both patient groups did not significantly differ statistically after Bonferroni correction (*p* > 0.0167). With 5.7 × 10^9^/L, the median WBC count in healthy controls was lower (*p* = 0.0014) than in SVT MPN+ patients but did not differ compared with the SVT MPN− group (*p* = 0.6218). With 132 vs. 139 g/L, median hemoglobin levels were slightly lower in the SVT MPN+ cohort than in the control group (*p* = 0.0101). Hemoglobin levels in the SVT MPN− cohort did not differ compared with the SVT MPN+ cohort and healthy controls. The hematocrit also did not differ between the three cohorts (Table 1).

### 2.2. Thrombin and APC Are Increased in Patients with MPN-Associated SVT

Table 2 shows measurement results of activation markers and other hemostasis parameters in the study population. Compared with international normalized ratio (INR) and APTT in healthy controls (median values of 1.0 and 26.4 s), INR and APTT were slightly higher, respectively, longer in the patient cohorts, with 1.2 (*p* = 1.8·10^−7^), respectively, 34.6 s (*p* = 2.8·10^−9^) in the SVT MPN+ group, and 1.0 (*p* = 0.0078), respectively, 29.9 s (*p* = 0.0027) in the SVT MPN− group, and they were higher, respectively, longer in the SVT MPN+ group than in the SVT MPN− group (*p* = 0.0064, respectively, *p* = 0.0066). Fibrinogen and antithrombin in the SVT MPN− cohort were higher, respectively, lower than in the control group, with median levels of 3.09 vs. 2.57 g/L (*p* = 0.0177) and 99 vs. 106% (*p* = 0.0028), respectively, but did not differ in these cohorts compared with the SVT MPN+ group (Table 2).

**Figure 2:**
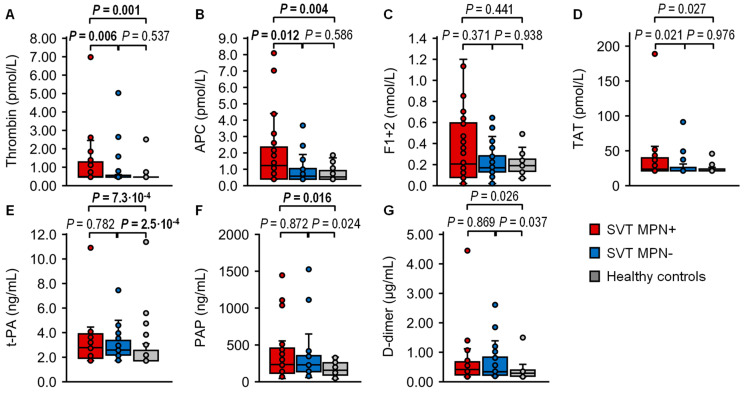
Activation markers. Plasma levels of (**A**) thrombin, (**B**) activated protein C (APC), (**C**) prothrombin fragment 1+2 (F1+2), (**D**) thrombin–antithrombin complex (TAT), (**E**) tissue-type plasminogen activator (t-PA) (**F**) plasmin-α2-antiplasmin complex (PAP), and (**G**) D-dimer were measured in patients with splanchnic vein thrombosis (SVT) with myeloproliferative neoplasms (MPN) (SVT MPN+, red, n = 36), patients with SVT without MPN (SVT MPN−, blue, n = 37), and healthy controls (gray, n = 30). Data are presented as box plots indicating quartiles and median of the data, the whiskers extending up to 1.5 times the interquartile range from the box, and circles showing outlying values. *p* values were calculated using the Kruskal–Wallis test followed by pairwise comparison using the Dunn procedure. Values of *p* ≤ 0.0167 were considered significant after Bonferroni correction for three comparisons and are shown in bold font.

Thrombin and APC in the SVT MPN+ cohort were increased compared with healthy controls, with median levels of 0.49 vs. <0.46 pmol/L (*p* = 0.0012), respectively, 1.23 vs. 0.54 pmol/L (*p* = 0.0035), and compared with the SVT MPN− cohort, with thrombin levels of 0.49 vs. <0.046 (*p* = 0.0057) and APC levels of 1.23 vs. 0.58 (*p* = 0.0122). Thrombin and APC in the SVT MPN− group did not differ compared with healthy controls (Table 2, Figure 2A,B). In contrast to free thrombin, plasma levels of the indirect thrombin markers prothrombin activation fragment 1+2 (F1+2) and the thrombin–antithrombin complex (TAT) did not significantly differ statistically between the three cohorts (Table 2, Figure 2C,D). Plasma levels of tissue-type plasminogen activator (t-PA) were increased in the SVT MPN+ and SVT MPN− cohorts compared with the control group, with median levels of 2.77 vs. <1.71 ng/mL (*p* = 7.3 × 10^−4^) and 2.59 vs. <1.71 ng/mL (*p* = 2.5 × 10^−4^) but did not differ between the two patient groups (Table 2, Figure 2E). With median levels of 234 vs. 156 ng/mL, plasmin-α2-antiplasmin complex (PAP) levels were higher in the SVT MPN+ cohort than in healthy controls (*p* = 0.0163) but did not differ statistically significantly between the SVT MPN+ and SVT MPN− cohorts and between the SVT MPN− cohort and the control group (Table 2, Figure 2F). D-Dimer levels also did not differ between the three groups (Table 2, Figure 2G).

**Figure 3:**
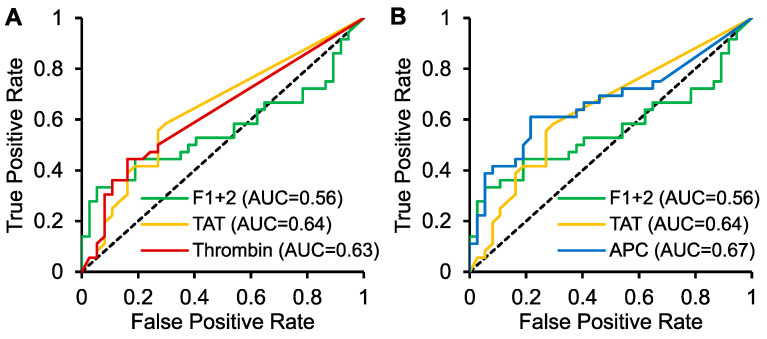
Receiver operating characteristics. The area under the curve (AUC) for (**A**) thrombin (red) and (**B**) activated protein C (APC, blue) is shown in comparison to prothrombin fragment 1+2 (F1+2, green) and thrombin–antithrombin complex (TAT, yellow).

To evaluate the diagnostic performance of the studied activation biomarkers of hemostasis in distinguishing SVT patients with MPN from those without MPN, receiver operating characteristic (ROC) analyses were performed. The results are presented in Figure 3. While the area under the curve (AUC) for thrombin was comparable to the AUCs for F1+2 and TAT (Figure 3A), APC showed a slightly higher AUC compared to these indirect biomarkers of thrombin formation (Figure 3B).

**Figure 4:**
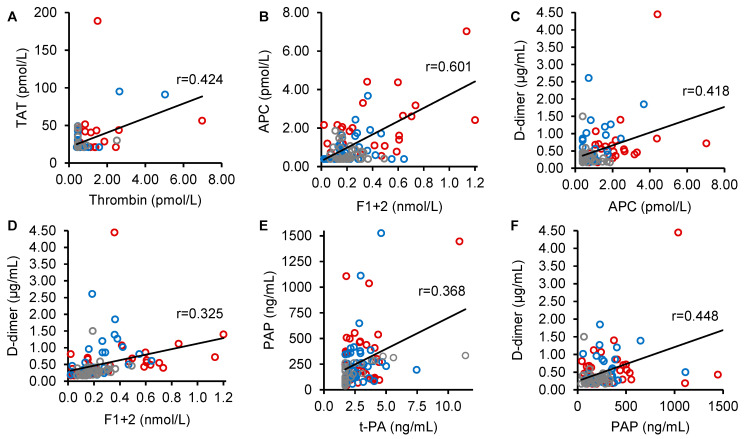
Statistically significant correlations between activation markers. Only correlations with *p* < 0.05 are shown to focus on relevant associations. Scatter plots of (**A**) thrombin and thrombin-antithrombin complex (TAT), (**B**) prothrombin fragment 1+2 (F1+2) and activated protein C (APC), (**C**) APC and D-dimer, (**D**) F1+2 and D-dimer, (**E**) tissue-type plasminogen activator (t-PA) and plasmin-α2-antiplasmin complex (PAP), and (**F**) PAP and D-dimer measured in patients with splanchnic vein thrombosis (SVT) with myeloproliferative neoplasms (MPN, red circles, n = 36), patients with SVT without MPN (blue circles, n = 37), and healthy controls (gray circles, n = 30). The black line shows the Pearson correlation between each pair of variables in the overall study population (*r*, correlation coefficient).

Pearson analysis of bivariate correlation between thrombin, APC, F1+2, TAT, t-PA, PAP, and D-dimer yielded statistically significant (*p* ≤ 0.0024) correlation coefficients for thrombin and TAT (*r* = 0.0424), F1+2 and APC (*r* = 0.601), APC and D-dimer (*r* = 0.418), F1+2 and D-dimer (*r* = 0.325), t-PA and PAP (*r* = 0.368), and PAP and D-dimer (*r* = 0.448) (Figure 4).

### 2.3. Association of Activation Marker Levels with Anticoagulant Treatment

With 83% in the SVT MPN+ cohort and 73% in the SVT MPN− cohort, most patients with SVT received anticoagulant treatment. Due to the heterogeneity of types and dosages of anticoagulant medications in the study population, patients on anticoagulation were grouped into the categories “full anticoagulation” and “reduced anticoagulation”, to analyze the effect of anticoagulant treatment on activation markers. The first group included patients on VKA (n = 13), apixaban 5 mg twice daily (BID) (n = 16), rivaroxaban 20 mg once daily (OD) (n = 10), dabigatran 150 mg BID (n = 2), edoxaban 60 mg OD (n = 1), and enoxaparin 1 mg/kg BID (n = 1). The group on reduced anticoagulation included patients receiving apixaban 2.5 mg BID (n = 8), rivaroxaban 10 mg OD (n = 7), enoxaparin 1 mg/kg OD (n = 3), and edoxaban 30 mg OD (n = 1).

**Figure 5:**
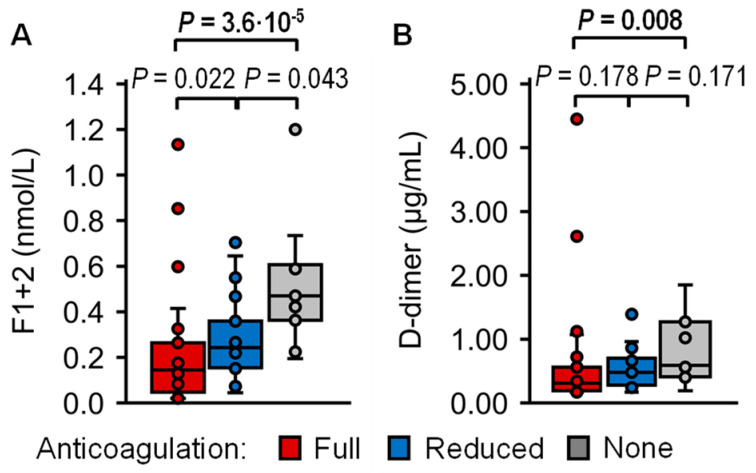
Activation marker differences associated with anticoagulant therapy. Plasma levels of (**A**) prothrombin fragment 1+2 (F1+2), and (**B**) D-dimer were measured in patients with splanchnic vein thrombosis with and without myeloproliferative neoplasms receiving full-dose (red, n = 43), reduced-dose (blue, n = 19), or no anticoagulant treatment (gray, n = 11). Data are presented as box plots indicating quartiles and median of the data, the whiskers extending up to 1.5 times the interquartile range from the box, and circles showing outlying values. *p* values were calculated using the Kruskal–Wallis test followed by pairwise comparison using the Dunn procedure. Values of *p* ≤ 0.0167 were considered significant after Bonferroni correction for three comparisons.

In patients on full dose anticoagulant treatment, plasma levels of F1+2 and D-dimer were significantly lower than in those without anticoagulant medication, with median levels of 0.15 vs. 0.47 nmol/L (*p* = 3.6·10^−5^) and 0.31 vs. 0.59 mg/L (*p* = 0.0084), respectively. F1+2 and D-dimer levels did not differ between patients on full anticoagulant treatment compared with patients on reduced anticoagulant treatment, and between patients on reduced anticoagulant treatment compared with those not on anticoagulation (Table 3, Figure 5A,B). Thrombin, APC, TAT, t-PA, and PAP did not differ between patients on full, reduced, and no anticoagulant treatment (Table 3, Figure S1).

## 3. Discussion

This is the first study that measured free thrombin and APC in patients with SVT. We found that MPN-positive patients, but not MPN-negative patients, with SVT were characterized by increased plasma levels of thrombin and APC. Increased plasma levels of APC like those observed here (1.23 pmol/L in SVT MPN+) have been reported previously in thrombophilic patients that carried the FV Leiden mutation (1.30 pmol/L [27] or the prothrombin 20210G>A mutation (1.03 pmol/L [28]). Furthermore, an association of the extent of APC formation rates with thrombosis history has been shown in FV Leiden carriers, i.e., in hereditary APC resistance [28]. An acquired APC resistance phenotype, associated with increased thrombin generation and a history of thrombosis, was previously observed in MPN [29,30]. Therefore, APC might be a biomarker candidate for the assessment of the hypercoagulable state in MPN.

In contrast to APC and indirect thrombin markers, increased plasma levels of free thrombin were not observed in the afore-mentioned previous studies, even when low-grade activation of coagulation was induced by recombinant activated factor VII (rFVIIa) [28,31]. Only in situations with a greater extent of coagulation activation, such as major surgery or septic shock, could increased plasma concentrations of free thrombin in the circulation be observed [31,32,33]. Plasma levels of coagulation activation markers are often viewed as global measures of hyper- or hypocoagulability. The most prominent example of this is D-dimer, when it is used to assess disseminated intravascular coagulation or to guide anticoagulant therapy [34,35,36]. Although being well-established and broadly applied, D-dimer is also a good example for demonstrating the difficulties in interpreting activation markers that arise from the different steps in dynamic pathways. For example, the major driver of increased D-dimer levels might be increased fibrinolysis, and not coagulation activation. Indeed, the presence of free thrombin and increased APC in the circulation of MPN-positive patients with SVT in this study might be indicative of more pronounced hypercoagulability in these patients than in patients with SVT without MPN, given that other demographic and clinical characteristics between the cohorts did not differ substantially. Observed differences in these characteristics between the MPN-positive and MPN-negative patient cohorts with SVT were either reflective of the specific clinical contexts of the respective cohorts (presence of somatic mutations and cytoreductive therapy in the SVT MPN+ cohort; liver disease, inflammatory bowel disease, infection/inflammation in the SVT MPN− cohort) or variations in blood count parameters within normal ranges. An alternative explanation for differences in thrombin and APC levels between cohorts could be differences in the pathomechanisms underlying the hypercoagulable states in SVT with and without MPN. The observed increase in thrombin levels in SVT MPN+ patients is possibly driven by the increased activation of platelets and leukocytes, release of procoagulant microparticles, or endothelial dysfunction associated with *JAK2 V617F*: This mutation has been shown to induce the expression of P-selectin on endothelial cells, which facilitates interactions between platelets, leukocytes, and the vessel wall and, thereby, increases thrombin generation [37,38]. Platelet-derived microparticles, which also show high prothrombinase activity, further contribute to this hypercoagulable state [39,40]. The simultaneous increase in APC levels indicates that the anticoagulant PC pathway remains intact despite the hypercoagulable state. The results of the ROC analysis, with a slightly higher AUC for APC, suggest a potential as a diagnostic marker for distinguishing SVT patients with MPN from those without.

Unlike thrombin and APC, the indirect thrombin markers F1+2 and TAT did not differ between the cohorts of patients with SVT and healthy controls. The comparison of TAT levels between cohorts revealed similar trends as were observed for thrombin, which did not, however, remain statistically significant after Bonferroni correction. These data disagree with previous studies that generally showed correlation between thrombin and indirect thrombin markers during surgery and in patients with septic shock [31,32,33]. Furthermore, increased levels of F1+2 and TAT in patients with MPN were observed previously [41]. This discrepancy for TAT may be partially explained by differences in the limits of detection (LOD), as a considerable number of patients showed plasma levels of thrombin and TAT below their respective LOD, which limits the visibility of anticoagulant treatment in these biomarkers. However, this explanation does not apply to F1+2, as most values for this biomarker were above the LOD. While anticoagulation probably contributed to lower F1+2 levels, the similar distribution of anticoagulant treatment of different intensities across cohorts suggests that this cannot explain why there were no significant differences between the SVT MPN+ and SVT MPN− cohort. Therefore, the discrepancy for F1+2 might instead reflect differences in thrombin generation dynamics. Increased levels of free thrombin may more directly indicate acute or localized hypercoagulability, while F1+2 may be influenced by additional factors, including anticoagulant treatment and other compensatory mechanisms, and hence be more reflective of a chronic hypercoagulable state. Moreover, circulating free thrombin may not fully represent in vivo thrombin generation, as a significant portion of thrombin is generated locally at the vessel and incorporated into the clot. Future studies are warranted to investigate the discrepancy between free thrombin, TAT, and F1+2 observed in this study and to better characterize the hypercoagulable state in SVT.

Like F1+2 and TAT, D-Dimer did not differ between cohorts, most probably also to the above-mentioned impact of anticoagulant treatment. With D-dimer being a compound activation marker of coagulation and fibrinolysis, the fibrinolysis biomarkers t-PA and PAP had been included in the panel of activation markers to allow for a better assessment of D-dimer levels. Both were increased in both patient cohorts with SVT in comparison to healthy controls, albeit PAP in the MPN-negative cohort only before Bonferroni correction, but they did not differ between MPN-positive and MPN-negative patients with SVT. Therefore, these markers might possibly be related to alterations in fibrinolysis accompanying SVT rather than being caused by (different) hypercoagulable alterations underlying the pathogenesis of SVT in MPN-positive and MPN-negative patients.

The strength of our study is the prospective collection of data, a well-characterized patient population, and the inclusion of a broad spectrum of activation markers of hemostasis and fibrinolysis. Limitations are that it is a single-center study with a small sample size, and that measurements of biomarker levels were not related to clinical endpoints. Therefore, the obtained results should be considered hypothesis-generating. The population was heterogeneous regarding the localization of SVT, the type of MPN, and the type and intensity of anticoagulant treatment. The considerable overlap in individual thrombin and APC values reflects the biological variability of SVT. While the observed differences were statistically significant at the cohort level, this overlap may limit their utility for individual diagnostics, warranting further investigation. Another limitation is that the interfering effect of anticoagulation hinders mechanistic interpretation of results with respect to underlying mechanisms of hypercoagulability.

## 4. Materials and Methods

This study was conducted from January 2022 to December 2023 at the University Hospital Bonn. The study procedures were performed in compliance with the approval granted by the Ethics Committee of the Medical Faculty at the University of Bonn on 24 July 2015 (protocol code 191/15) and adhered to the Declaration of Helsinki. Written informed consent was obtained from all participants, and the procedures were carried out in accordance with institutional guidelines.

### 4.1. Study Participants

Consecutive patients with SVT were recruited from referrals to the thrombophilia clinic of the Institute of Experimental Hematology and Transfusion Medicine. Healthy subjects were recruited from its blood donation service. While not all SVT patients within our catchment area are referred to our university hospital, there is no systematic exclusion of specific subgroups. Therefore, we believe our study population is broadly representative of the regional SVT patient population. After giving consent, patients with SVT and healthy controls aged 18 years or older were further assessed for eligibility. Patients were included if they had a personal history of SVT, defined as PVT, MVT, splenic vein thrombosis, or Budd–Chiari syndrome. Before they were referred to our clinic, they underwent a workup of MPN, including a complete blood count, peripheral blood smear review, molecular genetic analysis of MPN-associated clonal mutations (at least in JAK2, CALR, MPL genes), and, in case of abnormalities, a bone marrow biopsy. In all patients who were categorized as MPN, the diagnosis was confirmed by bone marrow biopsy, and patients with an incomplete workup of MPN were excluded. Further exclusion criteria were any other thrombosis or bleeding episode requiring treatment within two months prior to referral. Patients with SVT and healthy controls underwent thrombophilia testing, as described below, and healthy controls were excluded from the study if they showed any abnormalities. Thrombophilia was excluded in healthy controls to establish a baseline population without prothrombotic risk factors. In SVT patients, however, thrombophilia was not excluded to ensure that the patient cohorts represent the full spectrum of clinical presentations.

### 4.2. Reagents and Materials

The materials and devices used in this study are listed in Table S1. Blood samples were obtained, while patients were continuing their prescribed anticoagulant therapy, by puncture of a suitable vein using a 21 G or 23 G butterfly needle (Sarstedt, Nümbrecht, Germany). This approach was chosen to reflect real-world conditions and to ensure that biomarker levels were representative of the conditions under anticoagulant treatment. After discarding the first 2 mL, blood was drawn into citrate tubes (10.5 mmol/L final concentration). For thrombin measurement, citrate tubes additionally contained argatroban (100 µmol/L final concentration). For APC measurement, they additionally contained aprotinin (10 µmol/L final concentration) and bivalirudin (250 µg/mL final concentration). Plasma samples were obtained by centrifugation (2600× *g*, 10 min) within 30 min after the blood draw and stored at less than −70 °C before analysis.

### 4.3. Laboratory Analysis of Blood Samples

The OECAs for the measurement of thrombin and APC have been described elsewhere [27,42]. Studies in which they have been applied are listed in e.g., [43]. In brief, wells of microtiter modules were coated overnight at 4 °C with bovine serum albumin-biotin (10 µg/mL, 100 µL/well) and then washed. Streptavidin (10 µg/mL) was added to the wells and incubated for one hour at room temperature. After washing, 2% bovine serum albumin in phosphate-buffered saline, pH 7.4 were added to the wells and incubated for 2 h at room temperature for blocking. Subsequently to emptying the wells, biotinylated aptamers for capturing thrombin (HD1-22, 10 nmol/L) or APC (HS02-52G, 1 nmol/L) were added and incubated for 1 h at room temperature. Then, the plates were washed, and plasma samples for quantification, control samples, and plasma-based calibrators were placed in the wells. For the APC OECA, citrated plasma was recalcified using 1 mol/L CaCl_2_ (7.5 mmol/L final concentration). After incubation and subsequent washing, the fluorogenic peptide substrate I-1560 was added to the wells for the detection of thrombin. Pefafluor PCa was added for the detection of APC. Fluorescence over time was measured using a plate fluorescence reader. Samples were analyzed in triplicate. Calibrators covered a ½-log10 concentration range (0–272 pmol/L of thrombin or 0–182 pmol/L of recombinant APC (rAPC). The control samples consisted of primed (argatroban or aprotinin/bivalirudin) pooled normal plasma spiked with thrombin (13.6 and 136 pmol/L) or rAPC (9.1 and 91 pmol/L). Aliquots of the same controls were used in all runs. If one or more of the controls deviated more than 25% from target values, the run was repeated.

The assays used for all other laboratory analyses are listed in detail in Table S2. Thrombophilia testing included AT, PC, free PS, the dilute Russell viper venom time, lupus-sensitive and lupus-insensitive APTT, detection of anti-cardiolipin and anti-β2 glycoprotein I antibodies, as well as the FV Leiden and prothrombin 20210G>A mutations. Clotting times and clotting time-based assays, AT, PC, free PS, and D-dimer were determined using the Atellica Coag 360 coagulation analyzer and corresponding reagents (Siemens Healthineers, Erlangen, Germany). Concentrations of F1+2, TAT, PAP, and t-PA were measured using commercially available assays (Table S2). The LOD for TAT of 21.3 pmol/L was calculated based on the lowest validated standard in the calibration curve provided by the manufacturer (2 ng/mL) and assumes a molecular weight of approximately 94 kDa for TAT, derived from established values for thrombin and antithrombin [44,45]. Blood counts were performed using the Sysmex XN 1000 analyzer (Sysmex, Kobe, Japan).

### 4.4. Statistical Analysis

The normality of continuous data was tested using the Shapiro–Wilk test. Depending on normality, continuous data were compared using Student’s *t*-test, the Mann–Whitney test, or, if more than two groups were compared, the Kruskal–Wallis test followed by pairwise correction using the Dunn procedure. Frequency data were compared using the chi-square test, and, in case of cell frequencies below 5, Fisher’s exact test. Two-sided tests were used and values of *p* ≤ 0.05 were considered statistically significant. The Bonferroni method was used to correct for multiple testing. When comparing data between the three cohorts of (1) patients with SVT and MPN (SVT MPN+), (2) patients with SVT without MPN (SVT MPN−), and (3) healthy individuals (controls), values of *p* ≤ 0.0167 were considered statistically significant. Correlations were analyzed using the Pearson correlation coefficient. Values of *p* ≤ 0.0024 of correlation coefficients were considered statistically significant after correction for 21 comparisons using the Bonferroni method. All calculations were performed using the XLSTAT statistical and data analysis solution software, version 2021.4.1 (Addinsoft, Boston, MA, USA).

## 5. Conclusions

The obtained data suggest a potential role of free thrombin and APC as novel biomarkers for characterizing the hypercoagulable state in SVT patients, particularly those with MPN. In contrast, the changes observed in F1+2 levels with anticoagulant treatment suggest its potential as a marker to assess the efficacy of anticoagulation. Recent data suggest that plasma levels of D-dimer in patients on DOACs might differ from those in patients on VKA, challenging the validity of treatment decisions based on D-dimer levels alone [46,47,48]. One might speculate if especially free thrombin might be used in addition to D-dimer testing to guide anticoagulant treatment in patients with intense hypercoagulability such as MPN.

## Figures and Tables

**Table 1:** Characteristics of the study population and blood count parameters.

	SVT MPN+ (n = 36)	SVT MPN− (n = 37)	Controls (n = 30)	MPN+ vs. MPN−, *p*
Age in years	56 (29–80)	56 (32–72)	48 (29–67)	0.8335
vs. controls, *p*	**0.0082**	**0.0064**	-	-
Male sex	18 (50%)	21 (57%)	17 (57%)	0.5657
vs. controls, *p*	0.5902	1.0000	-	-
Body mass index, kg/m^2^	21.3 (19.7–28.3)	23.6 (21.1–26.5)	23.3 (22.0–24.6)	0.2302
vs. controls, *p*	0.4819	0.6630	-	-
Portal vein thrombosis *	35 (97%)	30 (81%)	-	0.0556
Splenic vein thrombosis	19 (53%)	14 (38%)	-	0.2003
Mesenteric vein thrombosis	13 (36%)	13 (35%)	-	0.9203
Months since SVT diagnosis	33 (19–90)	21 (7–42)	-	0.0969
Additional other thrombosis †	17 (47%)	12 (32%)	-	0.1963
Previous VTE	8 (22%)	7 (19%)	-	0.7290
Coronary artery disease	1 (3%)	5 (14%)	-	0.1992
MPN-associated mutation ‡	35 (97%)	1 (3%)	-	**1.6 × 10^−18^**
Malignancy other than MPN	6 (17%)	6 (16%)	-	>0.999
Thrombophilia	7 (19%) §	5 (14%) **	-	0.4930
Arterial hypertension	12 (33%)	9 (24%)	-	0.3961
Diabetes mellitus	6 (17%)	10 (27%)	-	0.2857
Liver disease	1 (3%)	12 (32%)	-	**9.3 × 10^−4^**
Inflammatory bowel disease	0	6 (16%)	-	**0.0251**
Other autoimmune disorder	4 (11%)	5 (14%)	-	>0.999
Other infection/inflammation	4 (11%)	12 (32%)	-	**0.0276**
Recent abdominal surgery	0	3 (8%)	-	0.2397
Full anticoagulation ††	21 (58%)	22 (59%)	-	0.9203
Reduced anticoagulation ‡‡	9 (25%)	10 (27%)	-	0.8415
Antiplatelet drugs §§	9 (25%)	3 (8%)	-	0.0640
Cytoreductive therapy	24 (67%)	-	-	**1.1 × 10^−10^**
Platelets, 10^9^/L	308 (226–365)	201 (313–290)	255 (217–301)	**1.8 × 10^−4^**
vs. controls, *p*	0.1670	0.0292	-	-
White blood cells, 10^9^/L	7.9 (5.4–11.0)	5.8 (4.6–7.5)	5.7 (4.9–6.1)	**0.0042**
vs. controls, *p*	**0.0014**	0.6218	-	-
Hemoglobin, g/L	132 (114–140)	134 (116–147)	139 (135–148)	0.3478
vs. controls, *p*	**0.0101**	0.0904	-	-
Hematocrit, %	40.9 (35.7–44.3)	39.4 (36.9–44.4)	40.6 (39.0–42.6)	0.7236
vs. controls, *p*	0.5964	0.8447	-	-

Age is presented as mean and range. All other continuous variables are summarized as median and quartiles. Dichotomous data are presented as counts and percentages. *p* values were calculated using Student’s *t*-test for age, the Kruskal–Wallis test followed by pairwise comparison using the Dunn procedure for other continuous data, the chi-square test for frequency data, and Fisher’s exact test for cell frequencies below 5. For continuous parameters and sex, values of *p* ≤ 0.0167 were considered significant after Bonferroni correction for three comparisons (*p* ≤ 0.05 for other parameters). *p* values considered significant are shown in bold font. * Thereof one patient with Budd–Chiari syndrome in the SVT MPN+ cohort, two in the SVT MPN− cohort. † History of any arterial or venous thrombosis including superficial vein thrombosis. ‡ Thereof, one CALR mutation in the SVT MPN+ cohort, JAK2 V617F in all other patients. § Heterozygous factor V Leiden (FVL) mutation (n = 5) or prothrombin 20210G>A (PTM) mutation (n = 1), antiphospholipid syndrome (n = 1). ** Heterozygous FVL (n = 3), Heterozygous FVL and PTM (n = 1), homozygous PTM (n = 1). †† Vitamin K antagonist, full dose low molecular weight heparin (LMWH), rivaroxaban 20 mg once daily, apixaban 5 mg twice daily, dabigatran 150 mg twice daily. ‡‡ LMWH or direct oral anticoagulants at lower doses. §§ Thereof one patient on clopidogrel in the SVT MPN+ cohort, aspirin in all other cases. MPN, myeloproliferative neoplasm; SVT, splanchnic vein thrombosis; VTE, venous thromboembolism.

**Table 2:** Basic hemostasis parameters and activation markers.

	SVT MPN+ (n = 36)	SVT MPN− (n = 37)	Controls (n = 30)	MPN+ vs. MPN−, *p*
INR	1.2 (1.1–1.5)	1.0 (1.0–1.3)	1.0 (1.0–1.1)	**0.0064**
vs. controls, *p*	**1.8 × 10^−7^**	**0.0078**	-	-
APTT, s	34.6 (30.0–38.1)	29.9 (26.4–32.0)	26.4 (25.7–27.4)	**0.0066**
vs. controls, *p*	**2.8 × 10^−9^**	**0.0027**	-	-
Fibrinogen, g/L	2.65 (2.06–3.05)	3.09 (2.42–3.85)	2.57 (2.27–2.88)	0.0169
vs. controls, *p*	0.8063	**0.0117**	-	-
Antithrombin, %	99 (89–108)	99 (79–105)	106 (97–111)	0.5084
vs. controls, *p*	0.0189	**0.0028**	-	-
Thrombin, pmol/L	0.49 (<0.46–1.19)	<0.46 (<0.46–0.54)	<0.46 (<0.46–<0.46)	**0.0057**
vs. controls, *p*	**0.0012**	0.5373	-	-
APC, pmol/L	1.23 (0.43–2.22)	0.58 (<0.39–1.02)	0.54 (<0.39–0.89)	**0.0122**
vs. controls, *p*	**0.0035**	0.5863	-	-
F1+2, nmol/L	0.21 (0.09–0.59)	0.17 (0.13–0.28)	0.19 (0.14–0.25)	0.3712
vs. controls, *p*	0.4414	0.9383	-	-
TAT, pmol/L	23.7 (<21.3–37.7)	<21.3 (<21.3–26.0)	<21.3 (<21.3–23.7)	0.0208
vs. controls, *p*	0.0265	0.9764	-	-
t-PA, ng/mL	2.77 (1.92–3.80)	2.59 (2.21–3.20)	<1.71 (<1.71–2.38)	0.7820
vs. controls, *p*	**7.3 × 10^−4^**	**2.5 × 10^−4^**	-	-
PAP, ng/mL	234 (117–444)	230 (141–351)	156 (94–249)	0.8724
vs. controls, *p*	**0.0163**	0.0236	-	-
D-dimer, mg/L	0.42 (0.24–0.67)	0.34 (0.24–0.81)	0.29 (0.19–0.39)	0.8687
vs. controls, *p*	0.0259	0.0372	-	-

Data are presented as median and quartiles. *p* values were calculated using the Kruskal–Wallis test followed by pairwise comparison using the Dunn procedure. Values of *p* ≤ 0.0167 were considered significant after Bonferroni correction for three comparisons. *p* values considered significant are shown in bold font. APC, activated protein C; APTT, activated partial thromboplastin time; F1+2; prothrombin fragment 1+2; INR, international normalized ration; MPN, myeloproliferative neoplasm; PAP, plasmin-α2-antiplasmin complex; SVT, splanchnic vein thrombosis; TAT, thrombin-antithrombin complex; t-PA, tissue-type plasminogen activator.

**Table 3:** Activation markers in patients with SVT according to anticoagulant treatment.

	Full Dose (n = 43)	Reduced Dose (n = 19)	None (n = 11)	Full vs. Reduced, *p*
Thrombin, pmol/L	<0.46 (<0.46–0.78)	<0.46 (<0.46–0.61)	<0.46 (<0.46–1.09)	0.4052
vs. none, *p*	0.8288	0.6800	-	-
APC, pmol/L	0.56 (<0.39–1.39)	1.02 (0.64–1.90)	1.07 (0.57–2.16)	0.2232
vs. none, *p*	0.2201	0.8354	-	-
F1+2, nmol/L	0.15 (0.07–0.23)	0.24 (0.16–0.33)	0.47 (0.37–0.60)	0.0222
vs. none, *p*	**3.6 × 10^−5^**	0.0431	-	-
TAT, pmol/L	<21.3 (<21.3–27.3)	<21.3 (<21.3–22.8)	26.0 (<21.3–37.3)	0.3710
vs. none, *p*	0.1911	0.0693	-	-
t-PA, ng/mL	2.47 (1.98–3.29)	2.95 (2.35–3.85)	2.29 (2.05–3.81)	0.1189
vs. none, *p*	0.8043	0.3613	-	-
PAP, ng/mL	241 (128–358)	227 (168–451)	227 (129–360)	0.5640
vs. none, *p*	0.9959	0.8043	-	-
D-dimer, mg/L	0.31 (0.19–0.53)	0.48 (0.29–0.68)	0.59 (0.42–1.15)	0.1778
vs. none, *p*	**0.0084**	0.1709	-	-

Data are presented as median and quartiles. *p* values were calculated using the Kruskal–Wallis test followed by a pairwise comparison using the Dunn procedure. Values of *p* ≤ 0.0167 were considered significant after Bonferroni correction for three comparisons and are shown in bold font. APC, activated protein C; F1+2; prothrombin fragment 1+2; PAP, plasmin-α2-antiplasmin complex; SVT, splanchnic vein thrombosis; TAT, thrombin–antithrombin complex; t-PA, tissue-type plasminogen activator.

## Data Availability

Data are contained within the article or Supplementary Material.

## Supplementary Materials

The following supporting information can be downloaded at: https://www.mdpi.com/article/10.3390/ijms26010292/s1.
