# Supplementary material for: Assessment of Hypercoagulability in Splanchnic Vein Thrombosis by Measurement of the Hemostasis Enzymes Thrombin and Activated Protein C
Source: Int J Mol Sci. 2024 Dec 31;26(1):292. doi: 10.3390/ijms26010292 (PMC11719462; doi:10.3390/ijms26010292)
Supplement: Supplementary file 1 [file ijms-26-00292-s001.zip › Figure S1.pdf]

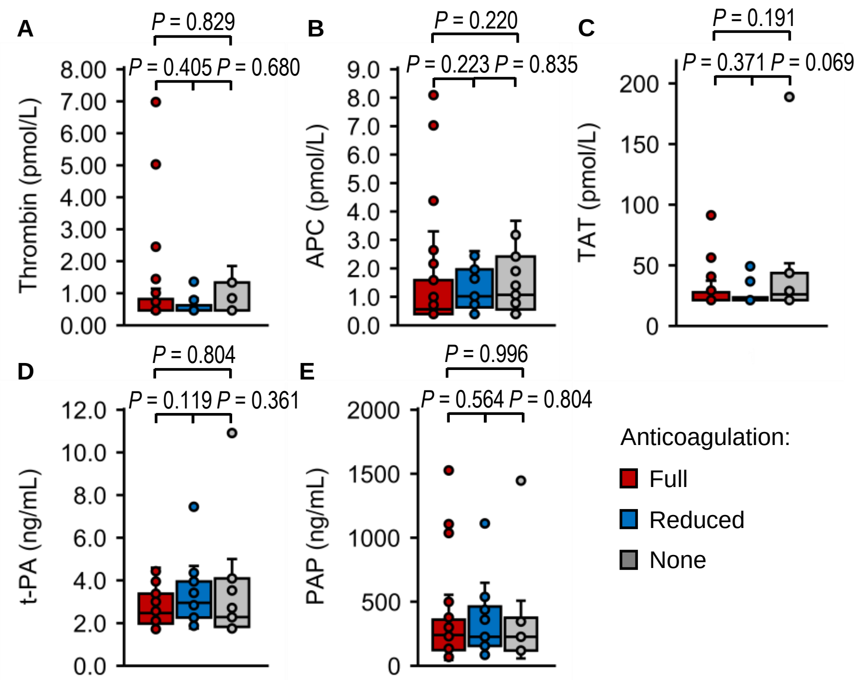

Figure S1. Activation marker levels according to anticoagulant therapy. Plasma levels of (**a**) thrombin, (**b**) activated protein C (APC), (**c**) thrombin-antithrombin complex (TAT), (**d**) tissue-type plasminogen activator (t-PA), and (**e**) plasmin-α2-antiplasmin complex (PAP) were measured in patients with splanchnic vein thrombosis with and without myeloproliferative neoplasms receiving full-dose (red, n=43), reduced-dose (blue, n=19), or no anticoagulant treatment (grey, n=11). Data are presented as box plots indicating quartiles and median of the data, the whiskers extending up to 1.5 times the interquartile range from the box, and circles showing outlying values. *P* values were calculated using the Kruskal-Wallis test followed by pairwise comparison using the Dunn procedure. Values of $P \leq 0.0167$ were considered significant after Bonferroni correction for three comparisons.
